# Supplementary material for: Pancreatic Cancer Organoids in the Field of Precision Medicine: A Review of Literature and Experience on Drug Sensitivity Testing with Multiple Readouts and Synergy Scoring
Source: Cancers (Basel). 2022 Jan 21;14(3):525. doi: 10.3390/cancers14030525 (PMC8833348; doi:10.3390/cancers14030525)
Supplement: Supplementary file 1 [file cancers-14-00525-s001.zip › Mäkinen et al., 2021 - Supplementary data 1.pptx]

## Slide 1
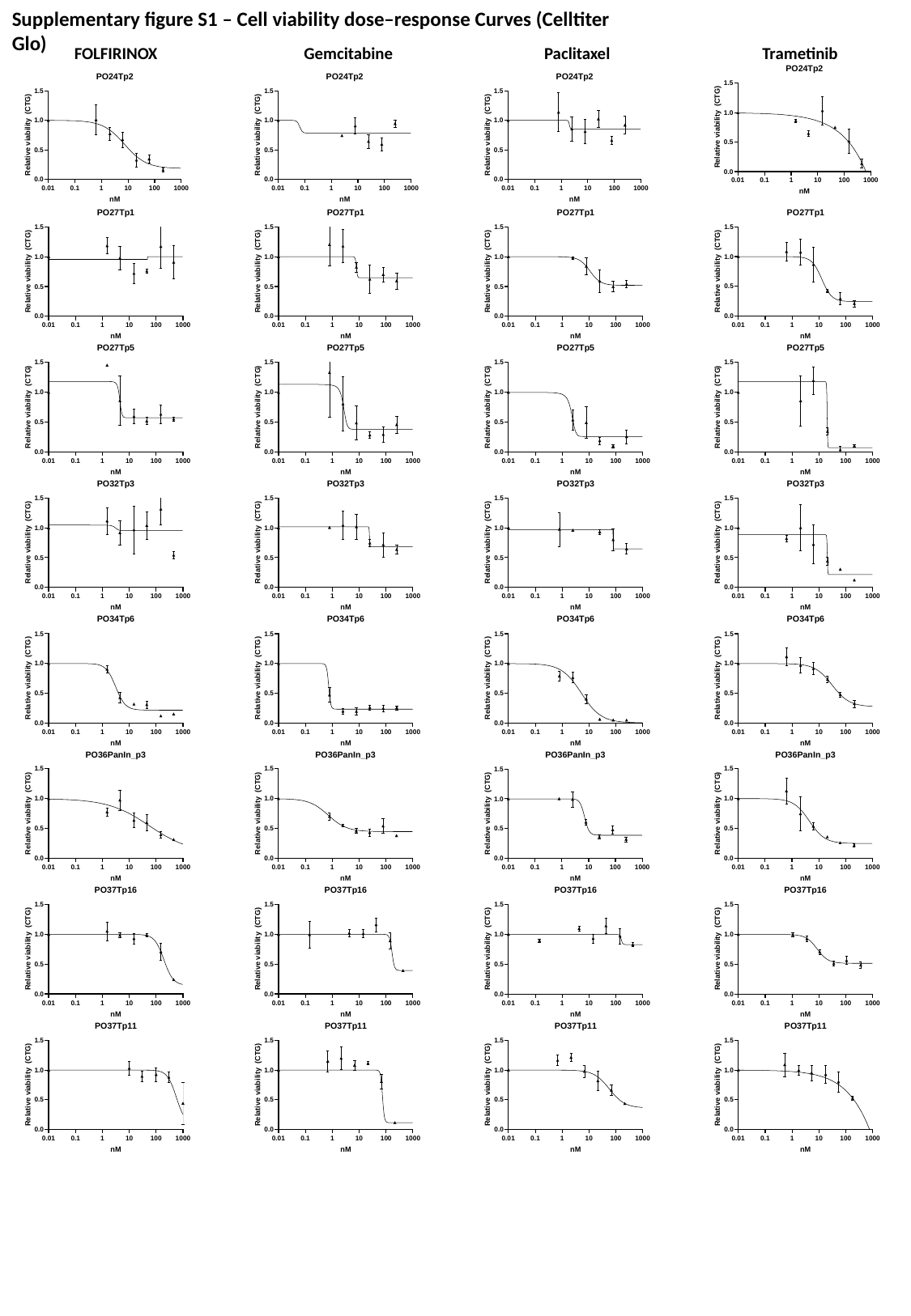

Supplementary figure S1 – Cell viability dose–response Curves (Celltiter Glo)
FOLFIRINOX
Gemcitabine
Paclitaxel
Trametinib

## Slide 2
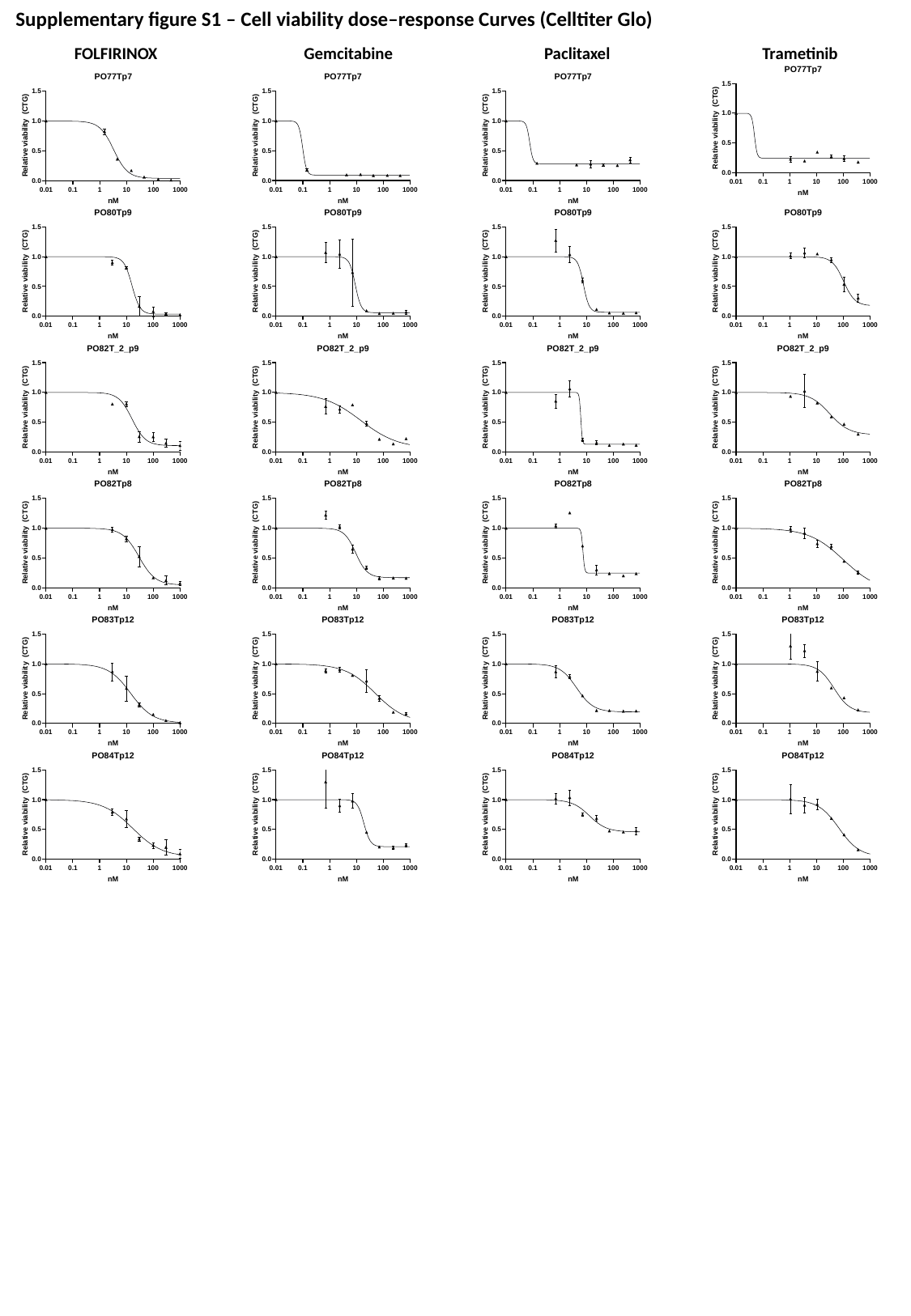

Supplementary figure S1 – Cell viability dose–response Curves (Celltiter Glo)
FOLFIRINOX
Gemcitabine
Paclitaxel
Trametinib
