# Supplementary material for: Pancreatic Cancer Organoids in the Field of Precision Medicine: A Review of Literature and Experience on Drug Sensitivity Testing with Multiple Readouts and Synergy Scoring
Source: Cancers (Basel). 2022 Jan 21;14(3):525. doi: 10.3390/cancers14030525 (PMC8833348; doi:10.3390/cancers14030525)
Supplement: Supplementary file 1 [file cancers-14-00525-s001.zip › Mäkinen et al., 2021 - Supplementary data 3.pptx]

## Slide 1
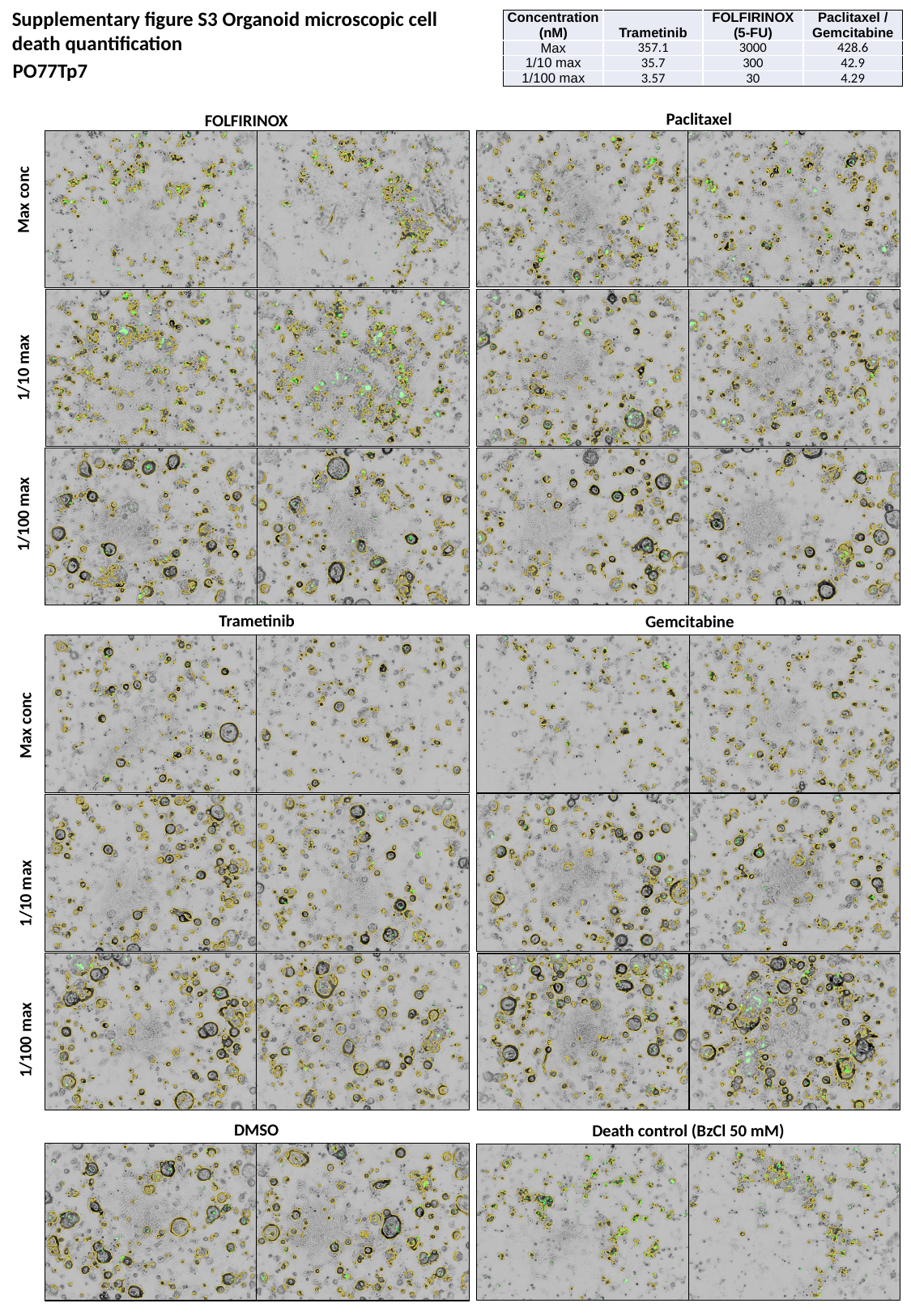

Supplementary figure S3 Organoid microscopic cell death quantification
| Concentration (nM) | Trametinib | FOLFIRINOX (5-FU) | Paclitaxel / Gemcitabine |
| --- | --- | --- | --- |
| Max | 357.1 | 3000 | 428.6 |
| 1/10 max | 35.7 | 300 | 42.9 |
| 1/100 max | 3.57 | 30 | 4.29 |
PO77Tp7
Paclitaxel
FOLFIRINOX
Max conc
1/10 max
1/100 max
Trametinib
Gemcitabine
Max conc
1/10 max
1/100 max
DMSO
Death control (BzCl 50 mM)
