# Supplementary material for: Pancreatic Cancer Organoids in the Field of Precision Medicine: A Review of Literature and Experience on Drug Sensitivity Testing with Multiple Readouts and Synergy Scoring
Source: Cancers (Basel). 2022 Jan 21;14(3):525. doi: 10.3390/cancers14030525 (PMC8833348; doi:10.3390/cancers14030525)
Supplement: Supplementary file 1 [file cancers-14-00525-s001.zip › Mäkinen et al., 2021 - Supplementary data 5.pptx]

## Slide 1
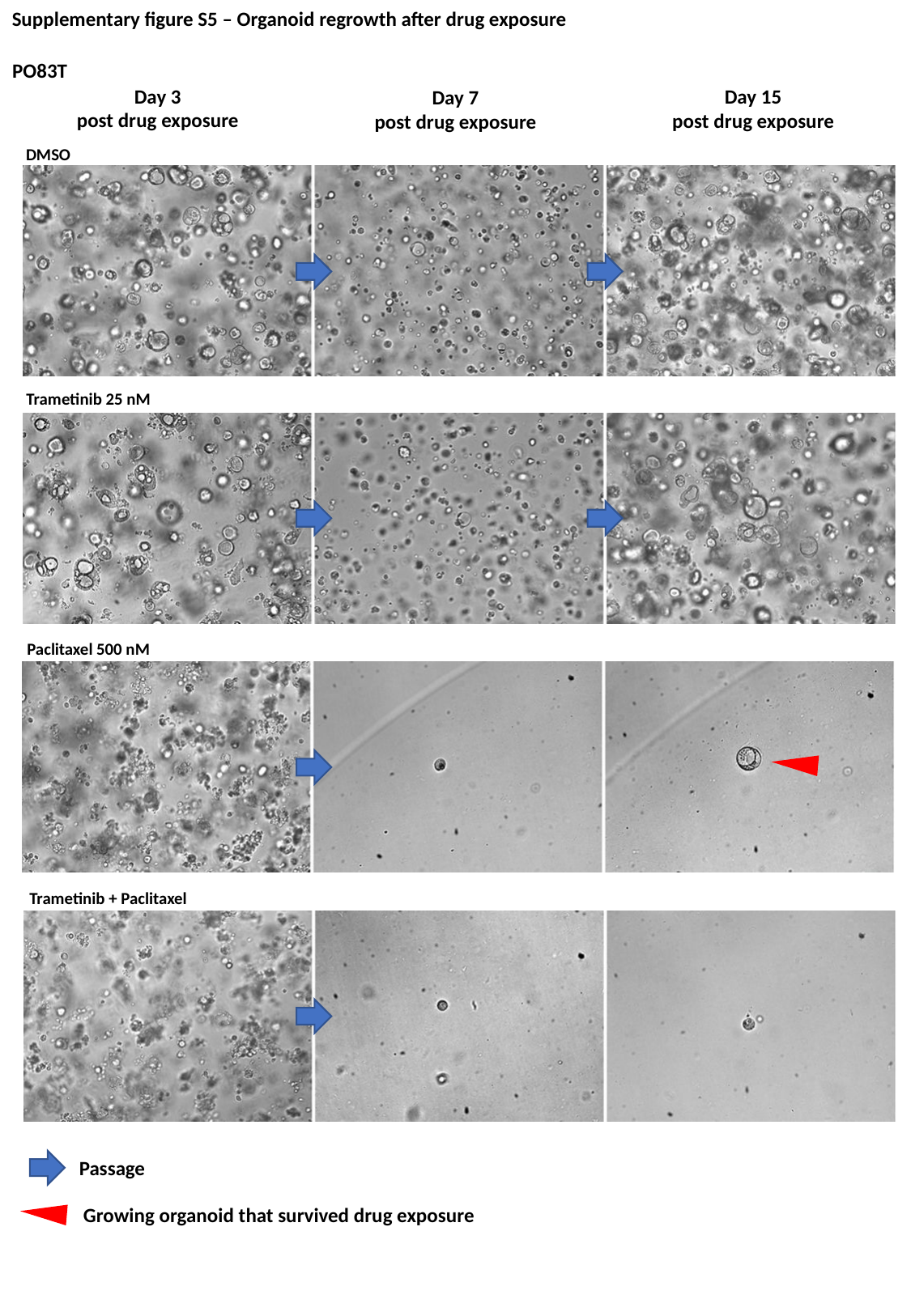

Supplementary figure S5 – Organoid regrowth after drug exposure
PO83T
Day 3
post drug exposure
Day 15
post drug exposure
Day 7
post drug exposure
DMSO
Trametinib 25 nM
Paclitaxel 500 nM
Trametinib + Paclitaxel
Passage
Growing organoid that survived drug exposure
